# Estimating net energy for activity for grazing beef cattle by integrating GPS tracking data, in-pasture weighing technology, and animal nutrition models

**DOI:** 10.3389/fvets.2025.1620584

**Published:** 2025-07-23

**Authors:** Logan Riley Vandermark, Jameson R. Brennan, Krista Ann Ehlert, Hector M. Menendez

**Affiliations:** ^1^Department of Animal Science, West River Research and Extension Center, South Dakota State University, Rapid, SD, United States; ^2^Department of Natural Resource Management, West River Research and Extension Center, South Dakota State University, Rapid, SD, United States

**Keywords:** precision livestock, animal nutrition, data integration, modeling, rangelands

## Abstract

Beef cattle production is largely dependent on rangelands for cattle to convert unusable plant-based fibers into an animal-based protein source for human consumption. Solutions are needed to meet both the growing demand for animal-based proteins and the desire of managers to produce energy-efficient cattle. Animal energetics has largely focused on beef cattle within confined systems such as feedlots. However, beef cattle grazing in extensive rangelands likely have a higher energetic requirement due to the need to forage across heterogeneous landscapes. In this study, we created a precision system model to account for net energy for activity of beef cattle on extensive rangeland systems by integrating in-pasture weighing technology, Global Positioning System (GPS) data, and animal nutrition models. The results from the mixed model analysis of variance (ANOVA) for net energy for maintenance activity (Nemr_act) indicated a significant main effect of treatment (*P* < 0.0001) and stocking rate (*P* < 0.0001), but there was no significant interaction (*P* = 0.705). These results indicate that, although the overall energetic expenditure may be similar, individual pasture effects may impact the proportional cost of physical activity partitioned between Resting, Flat, and Ascending energetic expenditures, as animals utilize diverse landscapes. Cattle grazing on extensive rangelands within the intermountain west with greater variations in both topography and slope will likely impact energetics to a greater extent. As the rates of precision technology and virtual fencing are adopted, the applications of the algorithm developed in this study may be used to quantify these differences at larger landscape scales across western rangelands.

## 1 Introduction

Beef cattle production in the western United States is largely dependent on rangelands. Cattle, within extensive production systems, often spend considerable amounts of energy traversing diverse landscapes to acquire their daily nutritional requirements through forage consumption. Although individual variations exist, on average, cattle travel approximately 7 km per day (1, 2), graze for approximately 7–9 h a day to meet dietary needs (3, 4), and rest for approximately 11 h a day (4). Cumulatively, these behaviors result in varying expenditures of energy both among individual animals and within the same animal on different days.

The Net Energy for maintenance (NEm) of beef cattle can be categorized into NEm requirements and NEm required for activity (NEmr_act). Beef cattle may depart from the modeled nutrient requirements due to continuous adaptation to stressors and the environment within extensive grazing systems (5). NEm is a measure of the amount of energy an animal needs to maintain its body weight, body temperature, and other basal metabolic functions while at rest. NEmr_act is the amount of energy that an animal needs to expend to capture resources through daily travel to food, water, and shelters across the landscape. Animal energetics has largely focused on animals in confined systems, such as feedlots or dry lots, for beef and dairy cattle. Consequently, rangeland cattle energetics are less known than those in confined systems. Cattle grazing on extensive systems likely expend more energy than animals in confined systems (6) because of the difference in the daily distance traveled. Other factors, such as topography, can also play an important role in the daily movement and behavior of grazing cattle (7). This can influence animal performance, as some animals are acclimatized or at a higher fitness level than others within a herd (6). There are unique challenges in determining the energetic expenditure for animals grazing on extensive rangeland systems, largely due to environmental factors and variations in topography (8).

Technological advancements in agriculture have enabled the opportunity to apply precision technology in rangeland cattle production systems (9). The implementation of radio frequency identification (RFID) tags, in-pasture weighing systems, and GPS tracking can provide a higher granularity of data that can be used to quantify animal energetics in extensive rangelands. As these technologies have become more widely adopted for livestock production, there are opportunities to utilize the resulting big datasets to improve animal efficiency and better determine energetic expenditure for grazing animals.

Energetic expenditure of grazing and walking was evaluated by Fox et al. (10) and later adapted by the NRC (11) to account for activity costs based on forage quality and quantity. A recent study by Tedeschi and Fox (12) proposed an equation to quantify NEmr_act based on animal movement metrics such as distance traveled on flat or ascending terrain, body weight, and time spent resting. Although the model has been used to estimate energetic expenditure at the herd level, no study has sought to incorporate precision weight and movement data to quantify NEmr_act at the individual level on a daily basis. Thus, the objectives of this study were to (1) develop a precision system model (PSM) that calculates daily NEmr_act for individual animals using GPS tracking collars and daily in-pasture weighing systems and(2) determine the impact of a virtually fenced rotational (VFR) grazing system vs. a continuous system on NEmr_act expenditure across three stocking rates for yearling steers grazing on Northern Mixed Grass Prairie.

## 2 Methods

### 2.1 Institutional animal care and use approval

The animal care and handling procedures used in this study were approved by the South Dakota State University (SDSU) Animal Care and Use Committee (Approval Number: 2104-021E).

### 2.2 Study area

This experiment was conducted at the SDSU Cottonwood Field Station (CFS), located in western South Dakota (43.9604, −101.8579). The CFS is located within a mixed-grass prairie ecosystem and is composed primarily of native C3 mid-grasses, including green needlegrass (*Nassella viridula* Trin.), needle-and-thread (*Hesperostipa Comata* Trin. & Rupr.), western wheatgrass (*Pascopyrum smithii* Rydb.), intermixed with native C4 short grass [blue gramma *Bouteloua gracilis* Willd. Ex Kunth, and buffalograss (*Bouteloua dactyloides* Nutt.)]. Recent introductions of non-native grasses, including Kentucky bluegrass (*Poa pratensis* Boivin & Love) and Japanese brome (*Bromus japonicus* Thunb.), are also prevalent at the site. The soil in the study area was predominately Kyle clay and Pierre clay (13). The topography was gently sloping with rolling hills and relatively flat-topped ridges, with a peak elevation of 784 m and a low elevation of 710 m. The climate was semi-arid, with hot summers and cold winters; annual precipitation for 2021 and 2022 was 278 mm and 267 mm, respectively (14). The long-term (1991–2020) average annual precipitation at the CFS is 452 mm (14).

### 2.3 Grazing management treatments

The study was overlaid on a long-term grazing study implemented in 1942 at the CFS on six pastures ranging in size from 31 to 73 ha (Table 1) (15). When the study was initiated, pasture boundaries were situated to uniformly allocate topographic features (hills, draws, ecological sites) across all stocking rate treatments. The long-term experimental design has been a randomized complete block with three levels of grazing intensity (light, moderate, and heavy) in two replicate blocks. Pastures in this study were stocked to maintain long-term stocking rate treatments: light (0.79 AUM/ha), moderate (0.99 AUM/ha), and heavy (1.78 AUM/ha).

**Table 1:** Estimates of pasture size and mean and standard deviation (SD) of elevation and slope for each of the six treatment pastures located at the South Dakota State University Cottonwood Field Station.

**Stocking rate**	**Grazing treatment**	**Pasture size (ha)**	**Mean elevation (m)**	**SD elevation (m)**	**Mean slope**	**SD slope**
Heavy	Continuous	31	734.4	5.83	2.29	1.25
Moderate	Virtual	54	741	6.61	3.37	1.58
Light	Virtual	65	745	7.78	2.96	1.54
Heavy	Virtual	31	741	6.97	2.89	1.68
Moderate	Continuous	53	752.6	8.05	3.28	1.65
Light	Continuous	73	752.9	9.926	3.57	1.6

Pastures were established in 1942 as part of a long-term stocking-rate study. A 10 m digital elevation map was utilized for calculating elevation and slope for each pasture.

Black Angus yearling steers (*n* = 127 and *n* = 135 in 2021 and 2022, respectively) were utilized in this study. In 2021, steers grazed between June 10 and August 17. In 2022, steers grazed between June 8 and August 21. Steers were allocated to two treatment groups, a continuous grazing treatment (CG) and a virtual fence rotation (VFR) treatment, across three stocking rates in a 2 × 3 factorial design. VFR steers were managed in a rotational grazing system using a Vence^TM^ virtual fencing system (Merck, Rahway, NJ). Steers within the VFR treatment were rotated among 3–4 virtual ‘paddocks' within the pastures for the duration of the grazing season. Days within each virtual fence rotation were determined based on forage availability sampled from biweekly clip plots for biomass estimation and calculated using the South Dakota State University Extension Grazing Calculator (16). Across both grazing management scenarios, VF collars were used to track animal locations at 5-min intervals; however, only animals within the VFR were managed with auditory and electrical cues enabled on the collars.

### 2.4 Weight data collection and processing

Daily individual steer weights were measured using SmartScales^TM^ (C-Lock Inc., Rapid City, SD, USA) in each pasture. SmartScales^TM^ is an in-pasture weighing technology that is placed in front of existing water tanks to measure animal body weight while drinking by recording RFID tag data and front-end weight, which is then converted to full body weight (17). Daily body weight data were downloaded via an application programming interface (API) (18). Spurious weights were removed from the dataset using a robust regression technique (19). For each animal, smoothing splines were fitted with body weight as the dependent variable and the day of the trial as the independent variable. Smoothing spline models were then used to predict daily body weight estimates for each steer, allowing for non-linear dynamics of animal growth, weight estimation on days when a valid weight was not recorded, and a reduction in the influence of gut fill on daily body weights. For model development, only animals with virtual fence collars retained for the duration of the season and with adequate weight data to estimate daily full body weight were utilized, resulting in 83 steers in 2021 and 53 steers in 2022, respectively.

### 2.5 Model development

The basis for this analysis was modified from an existing energetic equation developed from previously conducted research trials and empirically derived coefficients for determining NEmr_act (12). Equation 1 estimates NEmr_act (Mcals) from estimates of daily resting time (hours), number of state changes (e.g., changes from resting to grazing), daily horizontal travel distance on flat terrain (km), daily vertical ascending travel distance (km), and full body weight (FBW, kg). In their example, Tedeschi and Fox (12) determined NEmr_act expenditure at the herd level by determining the average slope of the pasture, average daily distance traveled (DDT), average weight, and average number of hours spent resting per day, with values varying based on the management system (i.e., confinement barn, conventional barn, dry lot, intensive grazing, and continuous grazing).


(1) $$\begin{array}{l} NEmr\_act=\\ \frac{{\left(\begin{array}{c} 0.1\;*resting\;time+0.062*number\;of\;state\;changes+0.621\\ \;*km\;flat\;travel+6.69*km\;ascending\;travel \end{array}\right)}^{*}FBW}{1000} \end{array}$$

An equation was developed to calculate the NEmr_act costs of beef cattle on rangelands. Where NEmr_act is Mcals expended per day, resting time is reported in hours per day, the number of state changes was held constant at 6 (based on the original equation), km of flat travel was reported as DDT where elevation change between successive GPS points was <1 meter of elevation difference, and km of ascending travel was reported as DDT where elevation change between successive GPS points was >1 meter of elevation difference (km of ascending travel derived in Equation 2, see below). The full body weight (FBW) is the weight of the animal.

In this study's adaptation of the model, inputs for Equation 1 were determined for individual animals daily by integrating metrics derived from SmartScales and Vence GPS location data, referred to as a precision systems model (20). To accomplish this, GPS data were first classified into grazing, resting, and walking behaviors based on the rate of travel (21). The total resting time for each day was calculated by summing the fixed duration (time between consecutive GPS points) for all locations classified as resting and converting it to hours for the variable “resting time” in the equation. Second, the daily distance traveled was partitioned into flat or ascending travel for each GPS point, classified as either grazing or walking. A 10 m digital elevation map (DEM) (22) was used to extract the elevation data (m) for each GPS point. Travel between successive fixes that were less than the absolute value of 1 m of elevation difference was defined as km of flat travel, and elevation differences greater than the absolute value of 1 m were classified as km of ascending travel. Movements associated with ascending or descending travel were grouped together as km of ascending travel. A previous study by Di Marco and Aello (23) showed no difference in energetic expenditure between ascending and descending walking in beef steers. Movement data, classified as km flat travel, were summed up to estimate the total daily km of travel distance. For GPS points classified as km of ascending travel, the vertical distance traveled was calculated as the absolute value of the elevation difference between consecutive GPS points. The vertical distance can also be derived trigonometrically using Equation 2, provided by Tedeschi and Fox (12). The total vertical distance traveled was summed for each individual steer daily and used as the estimate of km of ascending travel in Equation 1.


(2) $$\begin{array}{l} \text{k}\text{m}\;\text{ascending}\;\text{travel}=\\ \frac{{\left(\text{ascendin}{\text{g}}_{{\text{distance}}_{\text{a}}}-\text{ascendin}{\text{g}}_{{\text{distance}}_{\text{b}}}\right)}^{*}\cos\left({\text{inclination}}^{*}\frac{\text{p}\text{i}}{180}\right)}{\sin\left({\text{inclination}}^{*}\frac{\text{p}\text{i}}{180}\right)} \end{array}$$

Km of ascending travel can be calculated as the elevation difference between points A and B. Alternatively, this can be derived by taking the absolute value of the difference in elevation between consecutive GPS points from a digital elevation map.

This process resulted in both ascending and flat DDT for each steer. The model also accounted for the number of position changes per day; this value is the number of times an animal changed its behavior throughout the day (i.e., resting and walking). In our model, we used six as the number of position changes per day for animals under continuous and intensive grazing systems based on the values of Tedeschi and Fox (12). The final variable in the equation was the FBW (kg). Daily weights for each individual steer were estimated using SmartScales^TM^ as described above. The resulting output of Equation 1 provided a daily estimate of the individual steer NEmr_act (Mcals), which was converted into the metabolic rate of energy expenditure (kcal/BW^0.75^/d).

### 2.6 Statistical analysis

Daily estimates of the metabolic rate of energy expenditure (NEmr_act) were aggregated using weekly means for each pasture. In addition, each component of NEmr_act was calculated separately to estimate the relative contribution of resting energy expenditure (Resting EE), flat travel energy expenditure (Flat EE), and ascending travel energy expenditure (Ascending EE). The weekly mean for each energy expenditure component was calculated for each pasture and used for the analysis. The differences in NEmr_act, Resting EE, Flat EE, and Ascending EE between grazing treatments (VF and CG) and stocking rates (light, moderate, and heavy) were analyzed using linear mixed-effects model analysis of variance (ANOVA). Within the model, the fixed effects were stocking rate and treatment, with year and week specified as random effects. For significant main effects or interactions, *post-hoc* pairwise comparisons were conducted using Tukey's method, and least square means and standard errors were reported.

We performed a local and global sensitivity analysis [±10%, i.e., 90%, base (100%), and 110%] on NEmr_act in Vensim DSS (Ventana Systems™) using the following variables: resting (h/d), position change (number/d), flat travel (km/d), ascending travel (km/d), and BW (kg/d). The local calibration varied each variable one at a time for NEmr_act (Mcal/d) and also included evaluation of total NEmr (Mcal/d), while the global calibration varied all variables simultaneously using Latin Hypercube sampling and a multivariate distribution for a herd average NEmr_act. The local calibration produced daily individual NEmr_act values for each steer (*n* = 135), and the global calibration ran 100,000 simulations for the average daily NEmr_act from all steers. The data utilized to run the scenarios were driven by the observed data for all variables and included total digestible nutrients (TDN) derived from remote sensing algorithms, which were imported into Vensim from Excel. The estimated NEmr_act (Mcal/day) was used to estimate the daily rates of gain (kg/d) (24). When estimating the daily rates of gain, the 0.077 coefficient for NEmr was reduced by 10% to avoid double accounting, and then the estimated NEmr_act was added back to the total NEmr. A global regression was then run on the estimated BW and observed BW for each steer to assess the fit between simulation runs for energy corrected for activity (i.e., NEmr_act).

## 3 Results

Results from the mixed-model ANOVA for total NEmr_act energy expenditure indicate a significant main effect of treatment (*P* < 0.0001) and stocking rate (*P* < 0.0001), but no significant interaction (*P* = 0.705). Results indicate that steers with a heavy stocking rate had higher energetic expenditure compared to the light and moderate stocking rates (Table 2). Overall, animals with a heavy stocking rate expended 3.7% more energy than the light graze stocking rate and 5.6% more energy than the moderate stocking rate. Animals within the VF rotational system had significantly higher energy expenditure compared to the continuous graze treatment, with animals in the VF rotation expending 16.4 ± 0.89 kcal/BW^0.75^/d compared to 15.6 ± 0.89 kcal/BW^0.75^/d in the continuous graze system, a 5% increase, respectively.

**Table 2:** Least square means (kcal/BW^0.75^/d) followed by standard errors for net energy for maintenance activity (Nemr_act) and resting energetic expenditure (Rest EE) for steers grazing in the Northern Great Plains rangelands under three stocking rate intensities.

**Stocking rate**	**Nemr_Act**	**Rest EE**
Heavy	16.5^A^ ± 0.89	5.57^A^ ± 0.11
Moderate	15.6^B^ ± 0.89	5.96^B^ ± 0.11
Light	15.9^B^ ± 0.89	5.86^B^ ± 0.11

Energetic expenditure was calculated by integrating the GPS tracking data and in-pasture weighing technology with animal nutrition models. Different letters within columns indicate significant differences (*P* < 0.05).

**Figure 1:**
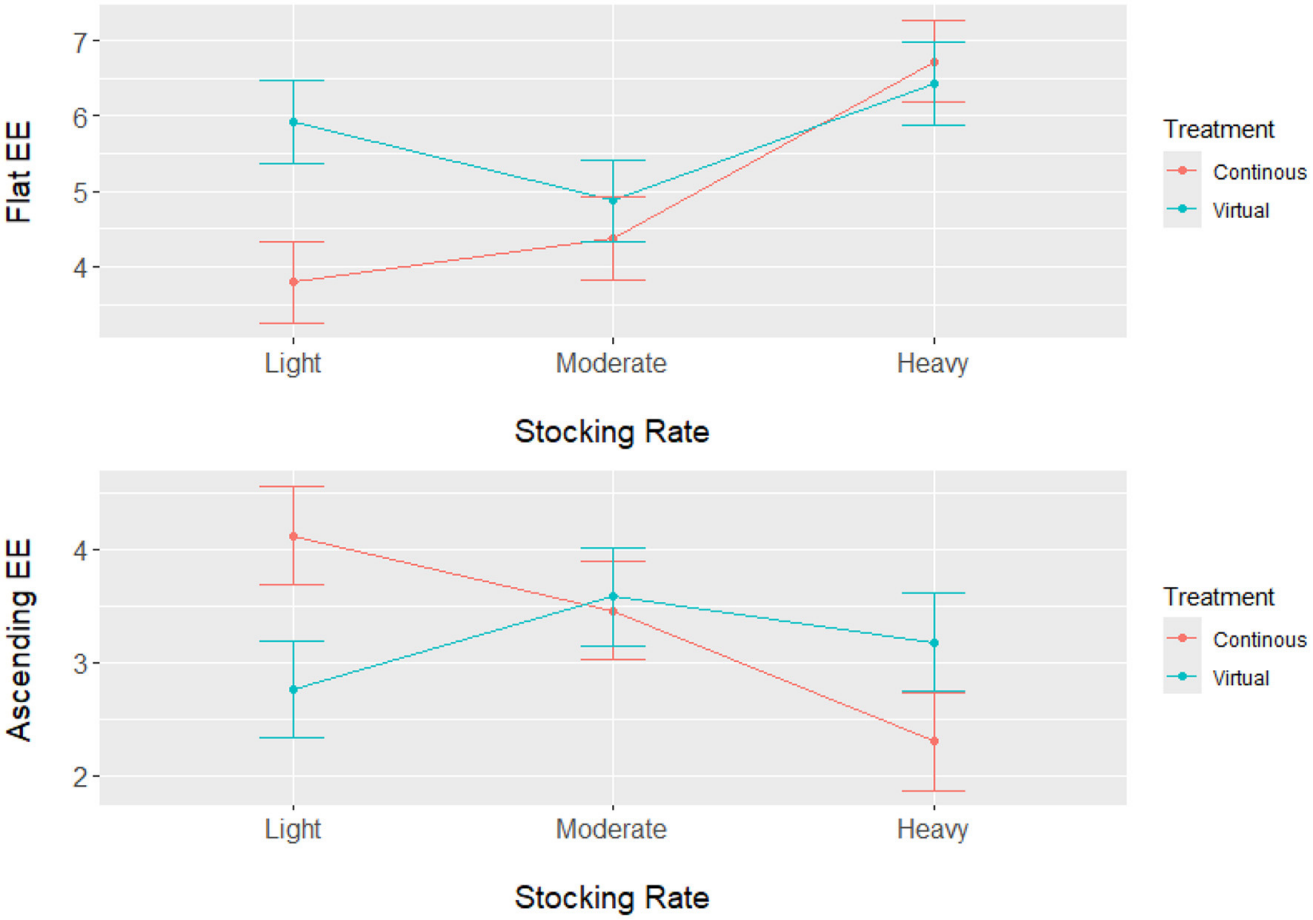
Least square means (kcal/BW^0.75^/d) with standard error bars for Flat Energetic Expenditure (Flat EE) and Ascending Energetic Expenditure (Ascending EE) for steers grazing in the Northern Great Plains rangelands under three stocking rate intensities and two grazing treatments (continuous and virtual rotation). Energetic expenditure was calculated by integrating GPS tracking data and in-pasture weighing technology with animal nutrition models.

Analysis of the energetic expenditure components of the model shows Resting EE had a significant main effect on stocking rate (*P* < 0.0001), but no significant impact of grazing treatment (*P* = 0.108) or interaction (*P* = 0.5211) between grazing treatment and stocking rate. Results from Resting EE show that the light and moderate stocking rates had significantly higher Resting EE compared with the heavy stocking rate (Table 2). These results indicate that the heavy stocking rate had a higher overall energetic expenditure for activity, but Resting EE was a lower component of the total NEmr_act compared to the light and moderate stocking rates. For the Flat EE and Ascending EE, both components of NEmr_act had a significant interaction (*P* < 0.0001) between stocking rate and grazing treatment. Least square means and standard errors from Flat EE and Ascending EE can be seen in Figure 1. Overall, the heavy stocking rate under both grazing treatments had the highest Flat EE, followed by the light virtual rate. The light stocking rate under continuous grazing had the lowest Flat EE (3.79 kcal/BW^0.75^/d) and the highest Ascending EE (4.12 kcal/BW^0.75^/d) of all treatment comparisons (Figure 1). These differences in the proportional contributions of Flat and Ascending EE may be due to differences in pasture topography, where the continuous light treatment had the highest mean elevation, standard deviation of elevation, and slope for all pastures (Table 1). These results indicate that although the overall energetic expenditure may be similar between treatment groups, individual pasture effects may impact the proportional cost of physical activity partitioned between Resting, Flat, and Ascending EE, as animals utilize diverse landscapes.

**Figure 2:**
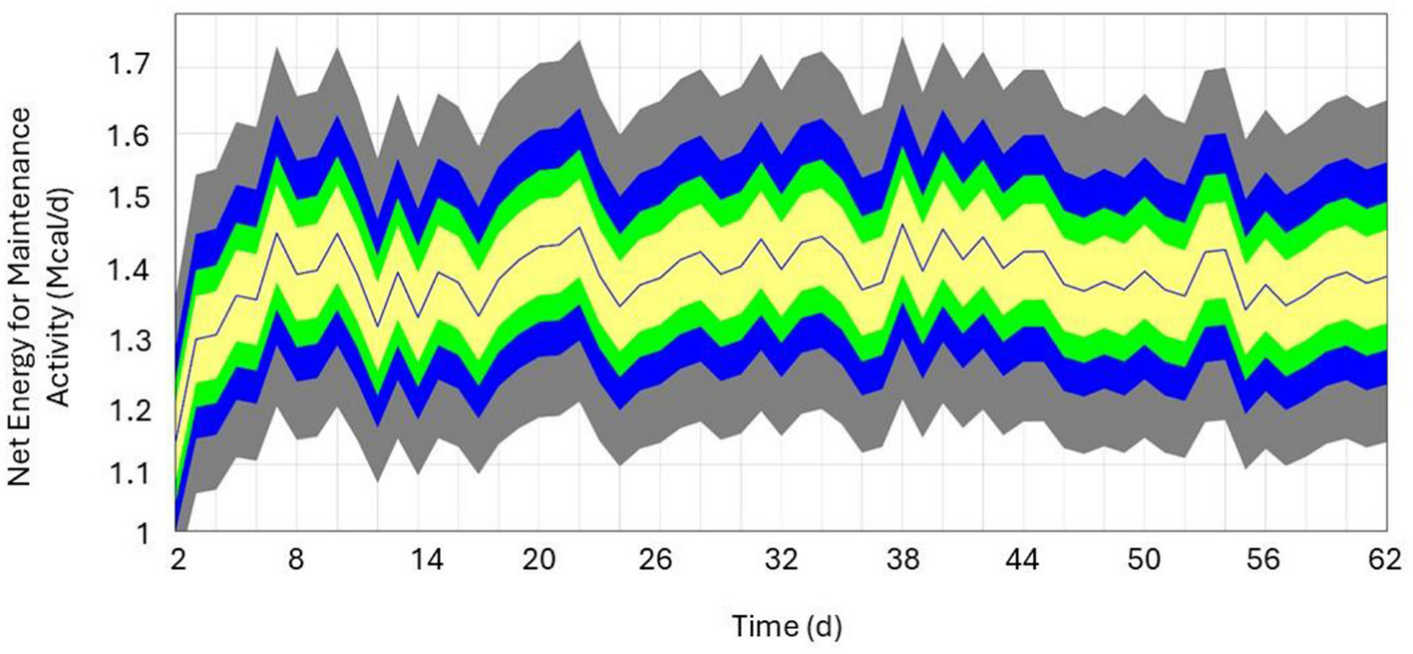
Sensitivity analysis of herd average Net Energy for Maintenance Required Activity (Mcal/d) over a 60-day period. Colors represent percentiles (yellow = 50%, green = 75%, blue = 95%, and gray = 100%), and the blue line represents the base run (i.e., model parameter values from the observed data).

**Figure 3:**
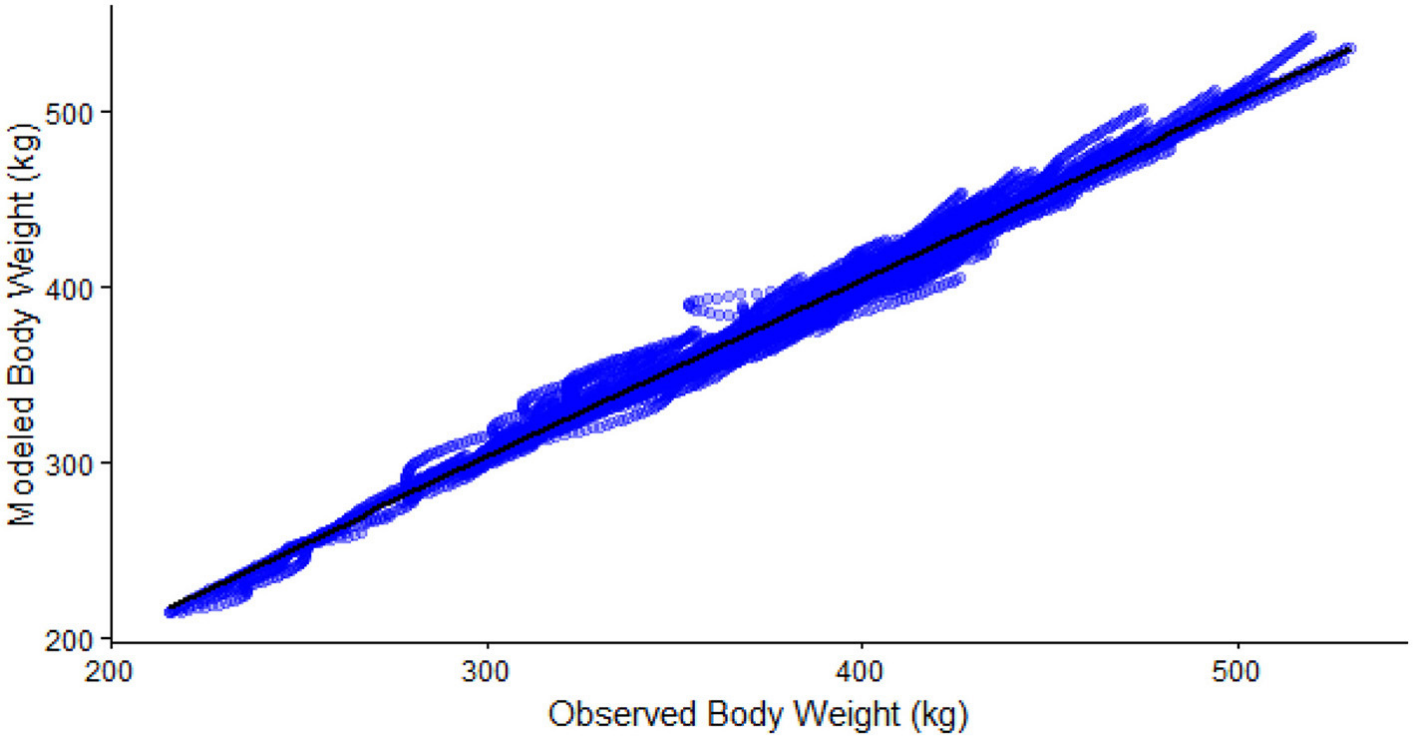
Regression of observed body weight (kg/hd/d) and predicted body weight (kg/hd/d, Adjusted R^2^ = 0.98).

The local sensitivity analysis resulted in a maximum change in NEmr_act from the base case (100%; Table 3) of 30%−33% less and 182%−199% greater for resting (h/d), position change, flat travel, vertical travel (km/d), and body weight (kg/d) when considering all individual animal's sensitivity from 90% to 110% scenarios. The local sensitivity analysis resulted in a maximum change in the total NEmr from the base case (100%; Table 4), 55%−60% less and 133%−143% greater for resting (h/d), position change, walking flat and vertical travel (km/d), and body weight (kg/d), respectively. Overall, NEmr_act and total NEmr were the least sensitive to position change and most sensitive to body weight. The global sensitivity resulted in an average range of 1 to 1.7 Mcal/d (Figure 2). The regression of predicted and observed body weights resulted in an adjusted R^2^ of 0.98 (Figure 3).

**Table 3:** Sensitivity analysis of NEmr activity from the base case (observed data) of individual steers (135).

**Variable**	**90%**	**100%**	**110%**
**Resting**			
Mean	1.33	1.38	1.43
Minimum	0.44	0.47	0.50
Maximum	2.46	2.49	2.52
Standard deviation	0.28	0.29	0.29
**Position change**			
Mean	1.36	1.38	1.39
Minimum	0.46	0.47	0.48
Maximum	2.47	2.49	2.51
Standard deviation	0.28	0.29	0.29
**Flat travel**			
Mean	1.33	1.38	1.42
Minimum	0.46	0.47	0.48
Maximum	2.34	2.49	2.64
Standard deviation	0.27	0.29	0.30
**Ascending travel**			
Mean	1.35	1.38	1.41
Minimum	0.46	0.47	0.48
Maximum	2.44	2.49	2.54
Standard deviation	0.28	0.29	0.29
**Body weight**			
Mean	1.24	1.38	1.52
Minimum	0.42	0.47	0.52
Maximum	2.24	2.49	2.74
Standard deviation	0.26	0.29	0.31

Values for resting (h/d), position change, flat travel, vertical travel (km/d), and body weight (kg/d) were adjusted to ±10%.

**Table 4:** Sensitivity analysis of total NEmr with estimated NEmr activity from the base case (observed data) for individual steers (135).

**Variable**	**90%**	**100%**	**110%**
**Resting**			
Mean	6.99	7.04	7.09
Minimum	4.17	4.19	4.22
Maximum	9.29	9.36	9.42
Standard deviation	0.95	0.96	0.96
**Position change**			
Mean	7.03	7.04	7.05
Minimum	4.19	4.19	4.20
Maximum	9.34	9.36	9.37
Standard deviation	0.95	0.96	0.96
**Flat travel**			
Mean	6.99	7.04	7.09
Minimum	4.19	4.19	4.20
Maximum	9.29	9.36	9.42
Standard deviation	0.94	0.96	0.97
**Ascending travel**			
Mean	7.01	7.04	7.07
Minimum	4.19	4.19	4.20
Maximum	9.31	9.36	9.43
Standard deviation	0.95	0.96	0.96
**Body weight**			
Mean	6.47	7.04	7.60
Minimum	3.86	4.19	4.52
Maximum	8.59	9.36	10.10
Standard deviation	0.88	0.96	1.03

Values for resting (h/d), position change, flat travel, vertical travel (km/d), and body weight (kg) were adjusted to ±10%.

## 4 Discussion

These results demonstrate a novel approach for integrating different technology data streams with animal nutrition models to estimate the energetic expenditure of animals grazing on rangelands. Energetic expenditure estimates from this approach are within the bounds of livestock physiology. In summarizing previous studies of energetic expenditure of cows on pasture, Tedeschi and Fox reported 11.7 kcal/BW^0.75^/d for standing behavior, 19.7 kcal/BW^0.75^/d for walking behavior, and 22.4 kcal/BW^0.75^/d for grazing behavior (12). These estimates are higher than our averages, which is likely due to differences in animal classes, where reported studies analyzed grazing activity in medium- and large-frame cows vs. yearling steers. Individual animal body weight is an important factor in calculating NEmr_act, where higher animal body weights equate to higher NEmr_act costs. This was further supported by the sensitivity analysis, which showed that FBW was the most influential factor on NEmr_act. Further, this is likely what drove the extremes in the minimum and maximum values in NEmr_act, across all animals with a range of body weights; some inherently have lower or higher NEmr_act. Given the increasing ability to assess or predict these metrics using precision livestock technologies and PSMs, more uniform classes of animals could allow managers to manage animal energetics more adequately relative to landscapes, resources, and distance to water.

Previous research has shown that traditional season-long grazing and adaptive rotational grazing management strategies do not impact energetic differences in yearling steers (25). However, Walker et al. (1) used calibrated pedometers and found that short-duration grazing animals traveled significantly more than continuous grazing animals. In a review of animal performance under continuous vs. rotational grazing systems, 92% of the studies reviewed had higher average daily gains for cattle under continuous vs. rotational grazing systems (26). Comparisons between traditional season-long grazing and adaptive rotational grazing systems showed that animals with a higher stock density in multi-paddock grazing systems had higher step counts later in the season, potentially in search of gut fill when forage quality and quantity were reduced (25). These results are similar to the results of this study, where animals within virtual rotations had higher overall energetic expenditure for yearling steers, potentially due to higher stock density within the virtual rotational system. In addition, our results indicate that stocking rates may impact the overall energetic expenditure. An assessment of steer performance in the northern mixed-grass prairie across different stocking rates reported that animals under heavy stocking rates had a 16% and 12% reduction in average daily gain compared with those under light and moderate stocking rates, respectively (27). Our results may point to a potential mechanism for this difference in performance, where animals within the heavy stocking rate had the highest total NEmr_act with the lowest proportion of Resting EE. In comparisons between continuous and rotational grazing systems, no difference was found in total resting time (28). Whereas previous research on sheep grazing systems demonstrated that as grazing intensity increases, the amount of time animals rested decreased and grazing time increased (29). These results were attributed to a reduction in forage biomass at higher grazing intensities, causing animals to increase grazing time to compensate for forage availability (29). These results agree with the finding that resting EE was not influenced by the grazing system but was influenced by the stocking rate. This may indicate that animals with heavy stocking rates partition energy differently by reducing their resting time, potentially because of increased competition for forage resources.

Other factors that can influence NEmr_act costs are genetics at both the individual and herd levels. Some animal breeds may travel farther from water and climb steeper gradients to forage (30). Energetic expenditure can vary based on animal genetics and the location where the cattle are grazing. Previous research has found that certain animals within a herd may utilize areas with greater elevation changes than others (31). Animals that travel more ascending/descending distances will likely increase grazing distribution within pastures, but potentially at a higher NEmr_act cost (30). Our results showed differences in ascending and flat energetic expenditure among treatments and across stocking rates. This may be because the individual pasture topography likely impacts the partitioning of flat vs. ascending travel. Factors such as topographic position class, elevation, and slope have been shown to influence the grazing distribution in the landscape (32, 33). While grazing selection has been shown to be influenced by topography, slope, and distance to water, travel between preferred grazing sites could influence energetic expenditure as animals traverse heterogeneous landscapes. Although the long-term experimental pastures used in this study were established to uniformly allocate topographic features (hills, draws, and ecological sites) across all stocking rate treatments, differences in the mean and standard deviation of elevation and slope still exist. This could explain why animals within the light continuous graze pasture had the lowest flat travel energetic expenditure and the highest ascending energetic expenditure, as it had the highest mean elevation and mean slope of the other pastures.

Pedometers have been used to calculate the daily distance traveled as a means to estimate energetic expenditure for rangeland cattle (1, 34–36). One benefit of using pedometers is that they may more accurately represent travel distance vs. GPS fixes, which could potentially underestimate travel due to meandering movements (37); however, pedometers fail to account for changes in elevation (25). With GPS technology, we can capture the location of an animal within a pasture and calculate the elevation changes associated with travel based on elevation maps. This may result in a more accurate estimation of energetic expenditure by portioning travel into flat or ascending travel to account for topography in energy estimates (8, 12, 38). GPS and pedometers in tandem can result in the most accurate classification of grazing, resting, and walking times (39). The combination of technologies, such as GPS, pedometers, accelerometers, and heart rate monitors, may provide a more accurate classification of movement behaviors across elevation gradients and subsequently better estimates of Nemr_act on rangeland systems.

Other factors, such as weather, can also influence animal energetics. The addition of climate data may also help refine energetic expenditure estimates of beef cattle in extensive systems. For example, extremely high temperatures result in heat stress, a factor known to increase energetic costs to regulate body temperature and maintain normal bodily functions (NRC 8^th^ edition). The temperature and humidity index (THI) has been used to determine the effects of weather on livestock energetics (40). Higher temperatures and subsequent heat loads on animals may also influence dry matter intake and daily distance traveled due to increased resting or loafing time near water (41). This would likely result in days with lower NEmr_act costs, increased NEmr costs to regulate body temperature, and a lower dry matter intake. The focus of this study was to provide a first step toward how precise livestock technology could be used to estimate NEmr_act in grazing beef steers. Future studies could seek to integrate real-time weather data into nutrition models to model the tradeoffs between behavior, dry matter intake, and NEmr for animals in extensive rangelands.

### 4.1 Management implications

This study developed a novel approach to estimate the energetic expenditure of beef cattle grazing on rangelands using metrics derived from precision livestock technology, geographic information system (GIS) analysis, and animal nutrition models. Virtual fence adoption has increased dramatically across the United States, where tens of thousands of animals are currently being tracked across diverse landscapes (42). Daily NEmr_act estimates coupled with genetic data may be used to identify cattle that are more efficient within a specific ecoregion. Genetics also creates variation in animals and their performance; this variation is not independent of location. Cattle grazing extensive rangelands within the intermountain west with greater variations in both topography and slope will likely impact energetics to a greater extent. The potential exists to leverage big datasets generated from these technologies to build regional energetic expenditure models to better predict livestock performance and inform the nutritional management of extensive rangelands.

## Data Availability

The raw data supporting the conclusions of this article will be made available by the authors, without undue reservation.
